# Wild-Type Measles Viruses with Non-Standard Genome Lengths

**DOI:** 10.1371/journal.pone.0095470

**Published:** 2014-04-18

**Authors:** Bettina Bankamp, Chunyu Liu, Pierre Rivailler, Jayati Bera, Susmita Shrivastava, Ewen F. Kirkness, William J. Bellini, Paul A. Rota

**Affiliations:** 1 Measles, Mumps, Rubella and Herpes Virus Laboratory Branch, Centers for Disease Control and Prevention, Atlanta, Georgia, United States of America; 2 Institute of Pathogen Biology, Chinese Academy of Medical Science & Peking Union Medical College, Beijing, People's Republic of China; 3 J. Craig Venter Institute, Rockville, Maryland, United States of America; University of Missouri-Columbia, United States of America

## Abstract

The length of the single stranded, negative sense RNA genome of measles virus (MeV) is highly conserved at 15,894 nucleotides (nt). MeVs can be grouped into 24 genotypes based on the highly variable 450 nucleotides coding for the carboxyl-terminus of the nucleocapsid protein (N-450). Here, we report the genomic sequences of 2 wild-type viral isolates of genotype D4 with genome lengths of 15,900 nt. Both genomes had a 7 nt insertion in the 3′ untranslated region (UTR) of the matrix (M) gene and a 1 nt deletion in the 5′ UTR of the fusion (F) gene. The net gain of 6 nt complies with the rule-of-six required for replication competency of the genomes of morbilliviruses. The insertions and deletion (indels) were confirmed in a patient sample that was the source of one of the viral isolates. The positions of the indels were identical in both viral isolates, even though epidemiological data and the 3 nt differences in N-450 between the two genomes suggested that the viruses represented separate chains of transmission. Identical indels were found in the M-F intergenic regions of 14 additional genotype D4 viral isolates that were imported into the US during 2007–2010. Viral isolates with and without indels produced plaques of similar size and replicated efficiently in A549/hSLAM and Vero/hSLAM cells. This is the first report of wild-type MeVs with genome lengths other than 15,894 nt and demonstrates that the length of the M-F UTR of wild-type MeVs is flexible.

## Introduction

**Figure 1:**
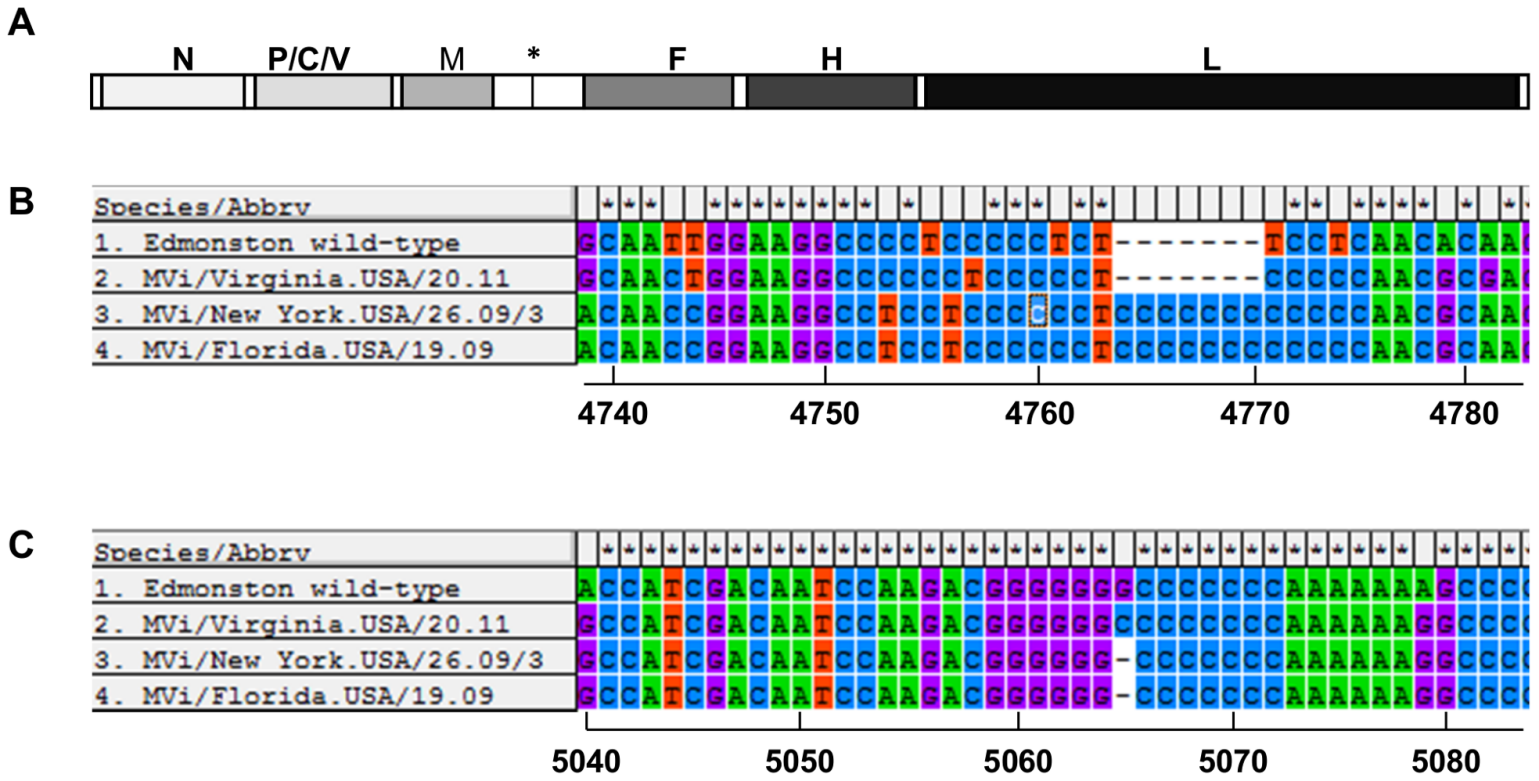
Location of insertions and deletions in the M-F UTR of MVi/New York.USA/26.09/3 and MVi/Florida.USA/19.09. A. Schematic representation of the MeV genome. The genome is depicted in the positive sense antigenomic orientation. Coding regions of each gene are in shades of grey, non-coding regions are white. Asterisk marks position of M-F intergenic trinucleotide at nucleotides 4872–4874. B. Insertion of 7 nucleotides in cytidine-rich region between nucleotides 4751–4775. C. Deletion of 1 nucleotide between nucleotides 5059–5072. The alignment was generated with MEGA5.10. Numbers under the alignment indicate nucleotide positions in aligned full-length genomes.

Measles virus (MeV) is a member of the genus *Morbillivirus* in the family *Paramyxoviridae*. Its non-segmented, negative sense RNA genome contains six genes separated by conserved intergenic trinucleotides [1]. In each gene, the coding regions are preceded and followed by untranslated regions (UTRs) (Fig. 1a), which include conserved transcription start and stop sequences, leading to the transcription of monocistronic mRNAs. Highly conserved promoter and encapsidation signals are located in the 52 nucleotide (nt) leader sequence and 37 nt trailer sequence at the termini of the genome. The size of the UTRs varies from 107–160 nt except for the 3′ UTR of the matrix protein (M) gene (positive sense antigenomic orientation) which is 426 nt and the 5′ UTR of the fusion protein (F) gene which is 583 nt long [2], [3]. The 1012 nt M-F UTR is highly variable and GC-rich, containing many homopolymeric sequences [1]–[4]. Experiments with plasmid-based transcription assays and recombinant MeVs indicated that the M-F UTR modulates the production of the M and F proteins [5]–[8]. The M protein lines the inside of the viral membrane and is responsible for tethering the nucleocapsid to the viral envelope. The F protein, consisting of subunits F1 and F2 is required for fusion of the virion with the host cell [9].

Experimental evidence with recombinant genomes demonstrated that the total length of the MeV genome must be divisible by six to produce a viable virus (the rule-of-six) [10]. While the standard length of the measles genome is 15,894 nt, conforming to the rule, there is abundant evidence from recombinant viruses that MeVs with genomes shorter or longer than 15,894 nt were able to replicate as long as they conformed to the rule of six [11]–[14]. However, the genomes for all non-recombinant MeV genomes published to date are 15,894 nt in length.

The genetic characterization of circulating wild-type MeVs is a critical component of laboratory surveillance for measles [15]. In combination with standard case classification, virologic surveillance provides a sensitive means to describe the transmission pathways of MeV. Virologic surveillance is required to document the interruption of transmission of endemic measles [16]. MeVs can be grouped into eight clades, subdivided into 24 genotypes based on the highly variable 450 nucleotides coding for the carboxyl-terminus of the nucleocapsid protein (N-450) [17], [18]. All vaccine strains belong to genotype A. However, N-450 does not always provide sufficient resolution to differentiate lineages within one genotype [19]. Differentiation of co-circulating lineages within a genotype may require expansion of the size of the region of the genome used for sequence analysis. While there is a large amount of sequence data available for the nucleocapsid protein (N) and hemagglutinin (H) genes for all genotypes (GenBank and Measles Nucleotide Surveillance database (MeaNS), www.who-measles.org), much less information is available for other regions of the genome. There is a programmatic need to increase the amount of sequence data available for the complete genomes of currently circulating, wild-type MeVs. Analysis of complete genomic sequences detected two wild-type MeVs with non-standard genome sizes.

## Methods

### RNA isolation, RT-PCR, 5′ RACE, and sequence analysis

The patient specimens analyzed in this study were submitted to CDC as part of routine surveillance of measles outbreaks in the USA. Specimens were anonymized and no patient records or associated metadata were included in the study. In such cases CDC does not require approval by an institutional review board. RNA was isolated from patient specimens using the QiaAmp Viral RNA Mini kit (Qiagen) and from lysates of infected cells with Trizol LS (Ambion). For sequencing of full genomes, oligonucleotide primers were designed using an automated primer design tool [20], [21]. Primers were designed from an alignment of the following reference sequences: AF266288, GQ376026, AB481088, AB481087, FJ416067, FJ416068, FJ211589, NC_001498, FJ161211, EF033071, EU293552, EU293550, DQ345721, EU435017, DQ227321, DQ211902, AY730614. Primers, with M13 tags added, were designed at intervals along both the sense and antisense strands, and provided amplicon coverage of at least 3-fold (Table S1). RT-PCR assays were performed with 1 ng of RNA using OneStep RT-PCR kits (Qiagen) according to manufacturer's instructions with minor modifications. Reactions were scaled down to 1/5 the recommended volumes, the RNA templates were denatured at 95°C for 5 min, and 1.6 U RNase Out (Invitrogen) was used. The RT-PCR products were sequenced with an ABI Prism BigDye v3.1 terminator cycle sequencing kit (Applied Biosystems). Raw sequence traces were trimmed to remove any primer-derived sequence as well as low quality sequence, and gene sequences were assembled using Minimus, part of the open-source AMOS project [The AMOS project. http://amos.sourceforge.net]. The gene sequences were then manually edited using CLOE (Closure Editor; JCVI) and ambiguous regions were resolved by additional sequencing when possible. The complete genomes were annotated with Viral Genome ORF Reader, VIGOR [22] before submitting to GenBank. VIGOR was used to check segment lengths, perform alignments, ensure the fidelity of open reading frames, correlate nucleotide polymorphisms with amino acid changes, and detect any potential sequencing errors.

**Figure 2:**
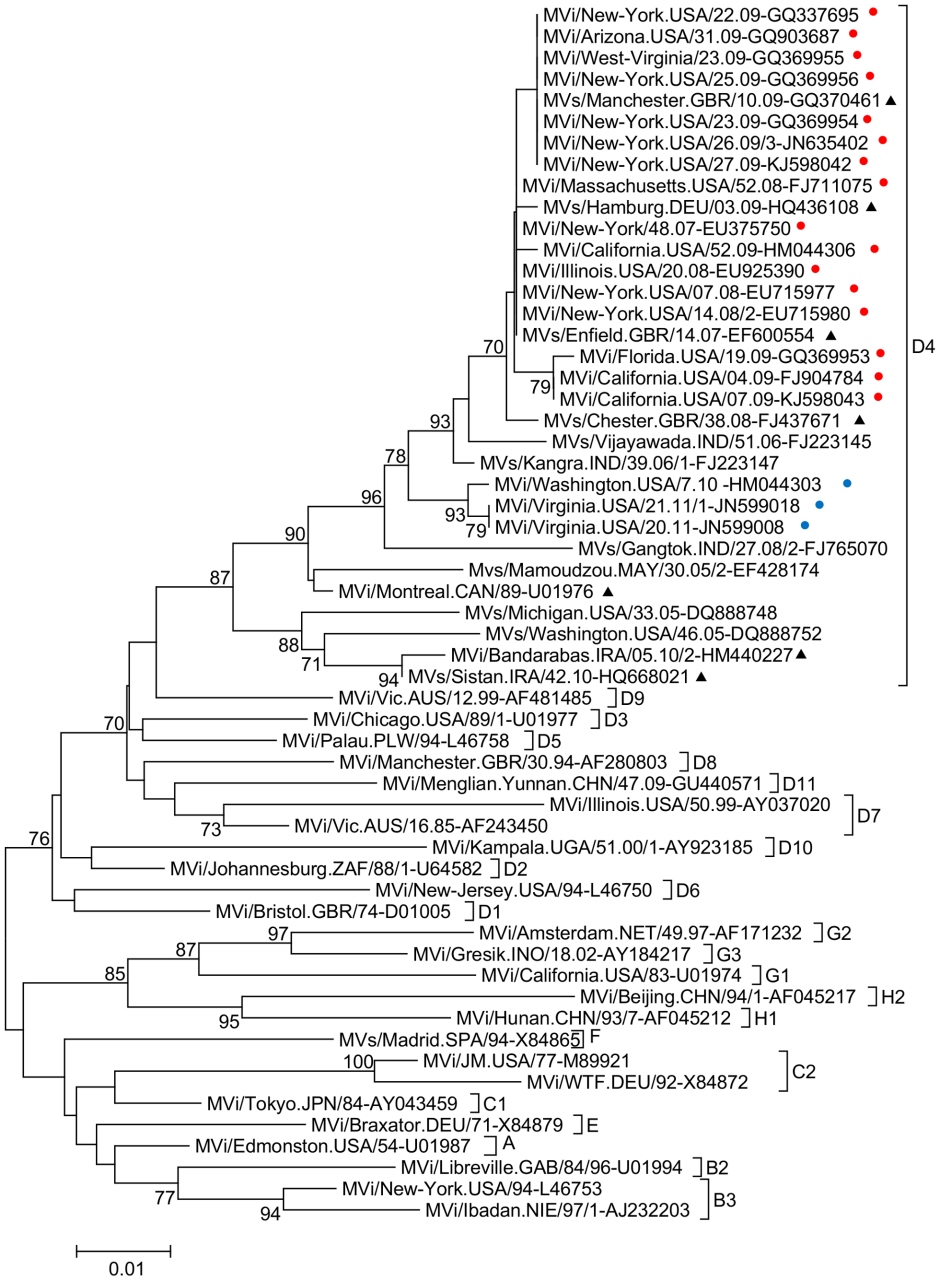
Neighbor joining phylogenetic tree based on the N-450 sequences from selected genotype D4 viruses and reference strains. Bootstrap values of at least 70% are shown. The scale bar shows the number of expected substitutions per site. Red dots indicate viruses sequenced in this study that share the indel in the M-F UTR; blue dots indicate viruses sequenced in this study that do not have the indel in the M-F UTR. Black triangles indicated named lineages in MeaNS.

The sequences of the genomic termini were obtained by using a 5′ RACE kit (Life Technologies). The PCR product representing the 5′ terminus of the genome (trailer) was sequenced directly. The PCR product representing the 5′ terminus of the antigenome (leader complement) was gel purified using a 3.5% NuSieve GTG agarose gel (Lonza) and the QiaQuick Gel Extraction kit (Qiagen). For sequencing of the M-F UTR, the region was amplified in one fragment (nt 4401–5558) or two fragments (nt 4401–4813 and 4884–5138). Reverse transcriptase reactions were performed using Superscript II reverse transcriptase (Life Technologies). PCR was performed with the Elongase enzyme mix (Life Technologies). PCR amplicons were purified with the PureLink PCR Purification kit (Life Technologies). Fragments were sequenced with the ABI Prism Big Dye Terminator v1.1 Cycle Sequencing kit (Applied Biosystems). The sequences of primers used for amplification and sequencing of the termini and the M-F UTR are available upon request. Sequences were analyzed using Sequencher v 5.1 (Gene Codes Corp.). Phylogenetic analysis was performed with MEGA v5.10 (megasoftware.net) using the maximum composite likelihood model. The reliability of phylogenetic inference at each branch node was estimated by the bootstrap method with 1000 replications. The full genomic sequences of MVi/New York.USA/26.09/3 and MVi/Florida.USA/19.09 were deposited in GenBank under accession numbers JN635402 and JN635403, under the bioproject id PRJNA69913. Partial sequences of M-F intergenic regions were submitted under accession numbers (KJ545611-KJ545627). Accession numbers for sequences used in Figure 2 are listed in the figure.

### Cell culture, plaque purification, virus stocks

Vero/hSLAM and A549/hSLAM cells (gift of Dr. Yanagi, Japan) [8], [23] were maintained in Dulbecco's modified Eagle's medium (DMEM) supplemented with 10% fetal bovine serum (FBS), glutamine, penicillin and streptomycin. 0.4 mg/mL G418 sulfate (Cellgro) was used to maintain expression of hSLAM in both cell lines. All viral isolates were obtained from the CDC strain bank. For MVi/New York.USA/26.09/3, MVi/Florida.USA/19.09, MVi/Washington.USA/07.10 and MVi/Virginia.USA/21.11/1, three rounds of plaque purification on Vero/hSLAM cells were performed. Individual plaques in plaque assays [24] were picked with sterile Pasteur pipettes, stored in 0.5 mL DMEM supplemented with 2% FBS, glutamine, penicillin and streptomycin (inoculation medium) at −70°C and used as inoculum for the next round of plaque purification. To grow virus stocks, cells in 25 cm^2^ flasks were infected with one plaque in inoculation medium and incubated until the cytopathic effect involved most of the monolayer. Cells were scraped into 1 mL of medium and frozen at −70°C. These small stocks were diluted 1∶2000 in inoculation medium and used to infect cells in 160 cm^2^ flasks. Stocks were harvested by scraping cells into 3 mL medium and frozen at −70°C. Thawed lysates were centrifuged at 4°C, 800 rcf, 5 min to produce cleared lysates. Virus titers were determined by plaque assay on Vero/hSLAM cells overlaid with inoculation medium containing 2% carboxyl methyl cellulose (MP Biomedicals).

### Multi-step growth curves

Vero/hSLAM and A549/hSLAM cells in 6 well culture plates were infected with virus stocks at an MOI of 0.001 in inoculation medium and incubated for 2 hours. Cells were washed with PBS and fresh inoculation medium was added. At different time points, cells were scraped into the medium and stored at −70°C. Titers of time course samples were measured by end-point titration in a 50% tissue culture infective dose (TCID_50_) assay. Ten-fold dilutions of the viral stock were prepared in inoculation medium and used to infect 6 wells of Vero/hSLAM cells per dilution in 48- well cell culture plates. Plates were incubated for 5 days and scored visually for the presence or absence of viral cytopathic effect. Titers were calculated using the Spearman-Kärber formula [25].

### Immunofluorescence assay

Vero/hSLAM and A549/hSLAM cells in chamber slides (LabTek II chamber slide system, Nalge Nunc International), were infected with 50 pfu/well in inoculation medium. Two hours after infection, the inoculum was replaced with inoculation medium containing 2% carboxyl methyl cellulose (MP Biomedicals) followed by incubation for 22 h. Cells were fixed with 3.7% formaldehyde (Fisher Scientific) and permeabilized with Triton-X-100 (Sigma-Aldrich). An anti-MeV nucleoprotein monoclonal antibody (83KKII, Millipore) was used for detection of infected cells, followed by an Oregon Green 488-conjugated secondary antibody (Life Technologies). Nuclei were counterstained with 1 mg/mL DAPI (Life Technologies). Fluorescence was visualized with a Zeiss Axio Imager.A1 microscope. An AxioCam MRc5 camera (Zeiss) and the Axiovision program v 4.8.2 were used for photography.

## Results

The complete genomic sequences of two measles isolates, MVi/New York.USA/26.09/3 and MVi/Florida.USA/19.09, were determined by Sanger sequencing and 5′ RACE. The genomes of both viral isolates were 15,900 nt in length, 6 nt longer than the standard length of the MeV genome. The increase in genome size was the result of an insertion of 7 nt in the 3′ UTR of the M gene and a deletion of 1 nt in the 5′ UTR of the F gene (Figure 1). Phylogenetic analysis based on N-450 determined that the genotype of both viral isolates was D4 (data not shown). Because there was no other full-length sequence of a genotype D4 virus with a standard genome organization available for comparison, all alignments were made using the Edmonston wild-type strain (AF266288, genotype A). Depending on the sequence of the genome used for comparison, the insertions may align differently. The insertions are located between nt 4751–4775, their exact position cannot be determined as the region is rich in homopolymeric sequences of cytidines. The deletion is located between nt 5059–5072, a region that contains homopolymeric sequences of guanidines and cytidines. Both the insertions and the deletion are located in non-coding regions of the genome and the net addition of 6 nt conforms with the rule-of-six [10]. To confirm that the indels were not the result of passaging the virus in cell culture, a PCR product spanning the M-F intergenic region was amplified from the patient sample of MVi/New York.USA/26.09/3. The insertions and deletion were confirmed in this PCR product. This result also makes it more likely that the indels were present in the viral genome, because the amplicon cannot be produced from M or F mRNAs. Amplification of the M-F intergenic region from sample MVi/Florida.USA/19.09 failed (Table 1).

**Table 1:** Detection of indels in the M-F UTR of viral isolates and patient samples.

	RNA source: virus isolate		RNA source: patient sample			
WHO Name	amplicon	indel	amplicon	indel	source of importation^a^	outbreak^b^
MVi/New York.USA/07.08	i	+	s	+	unknown	1
MVi/New York.USA/14.08/2	i	+	s	+	Belgium	1
MVi/New York.USA/48.07	i	+	s	+	Israel	2
MVi/Illinois.USA/20.08	i	+	af		unknown	3
**MVi/Florida.USA/19.09**	i	+	af		UK	4
**MVi/New York.USA/26.09/3**	i	+	i	+	unknown	5
MVi/New York.USA/22.09	i	+	s	+	unknown	5
MVi/New York.USA/23.09	i	+	s	+	unknown	5
MVi/New York.USA/25.09	i	+	s	+	unknown	5
MVi/New York.USA/27.09	i	+	af		unknown	5
MVi/California.USA/04.09	i	+	s	+	UK	6
MVi/California.USA/07.09	i	+	s	+	UK	6
MVi/West-Virginia.USA/23.09	i	+	af		unknown	7
MVi/Arizona.USA/31.09	i	+	af		France	8
MVi/Massachusetts.USA/52.08	i	+	s	+	India	9
MVi/California.USA/52.09	i	+	s	+	unknown	10
MVi/Washington.USA/07.10	nd		i	-	India	11
MVi/Virginia.USA/21.11/1	i	-^f^	af		India	12
MVi/Virginia.USA/20.11	i	-	af		India	12

a Source of importation based on epidemiologic investigation, ^b^arbitrary outbreak numbers; samples with the same outbreak number are in the same chain of transmission, i: sequence from single amplicon spanning M-F intergenic region, nd: sequence not done, +: indicates insertion and deletion present,: -: neither insertion or deletion present, s: sequence derived from separate amplicons for M and F UTRs, af: amplification failed, bold sample ID: complete genome sequenced.

While both viral isolates shared the same indels, their genomes differed in 3 nt positions in N-450 and 18 nt positions in the complete genomic sequence (data not shown). MVi/Florida.USA/19.09 was imported from the UK in May 2009, while MVi/New York.USA/26.09/3 was detected during a measles outbreak in New York with an unknown source of importation (Table 1). The epidemiological data and the sequence differences indicated that the two viral isolates were derived from separate chains of transmission.

To explore the frequency of the indels describe above, 16 additional viral isolates and one patient sample of genotype D4 from the CDC strain bank were examined for the presence of the indels (Table 1). These samples were collected from 2007 to 2011. Indels identical to those in MVi/New York.USA/26.09/3 and MVi/Florida.USA/19.09 were found in 14 viruses isolated from 2007–2010. Two viral isolates from 2011 and one patient sample from 2010 (MVi/Washington.USA/7.10) did not have indels in the sequenced region. The sequence of one isolate without the indels, MVi/Virginia.USA/20.11, is included in Figure 1. For all viral isolates and patient sample MVi/Washington.USA/7.10, sequence information was derived from PCR amplicons spanning the M-F intergenic region. Further support that the indels were not the result of passaging in cell culture was provided by detection of their presence in PCR products amplified from patient samples of 11 of the cases (including MVi/New York.USA/26.09/3). Confirmation in the other patient samples failed because of an insufficient quantity of clinical material. Because patient samples often do not contain enough intact genomic RNA to produce amplicons spanning the M-F intergenic region, separate PCR products for the M UTR and the F UTR were used to verify the presence of the indels. These data demonstrated that the indels were present in the original patient sample and are not a result of cell culture passage.

Epidemiological data (Table 1) indicated that the 16 cases with indels identified in the US can be grouped into 10 different imported chains of transmission. Where the source of infection was known, it was mostly Europe, with only one importation traced to India. The three cases without indels in the sequenced region belonged to two chains of transmission which were imported from India. A phylogenetic tree based on N-450 of all genotype D4 viruses studied in this report as well as reference sequences and other relevant genotype D4 sequences demonstrated that genotype D4 viruses group into distinct lineages corresponding to the presence or absence of indels (Figure 2). All samples with indels are closely related to the Manchester and Enfield named lineages [18].

**Figure 3:**
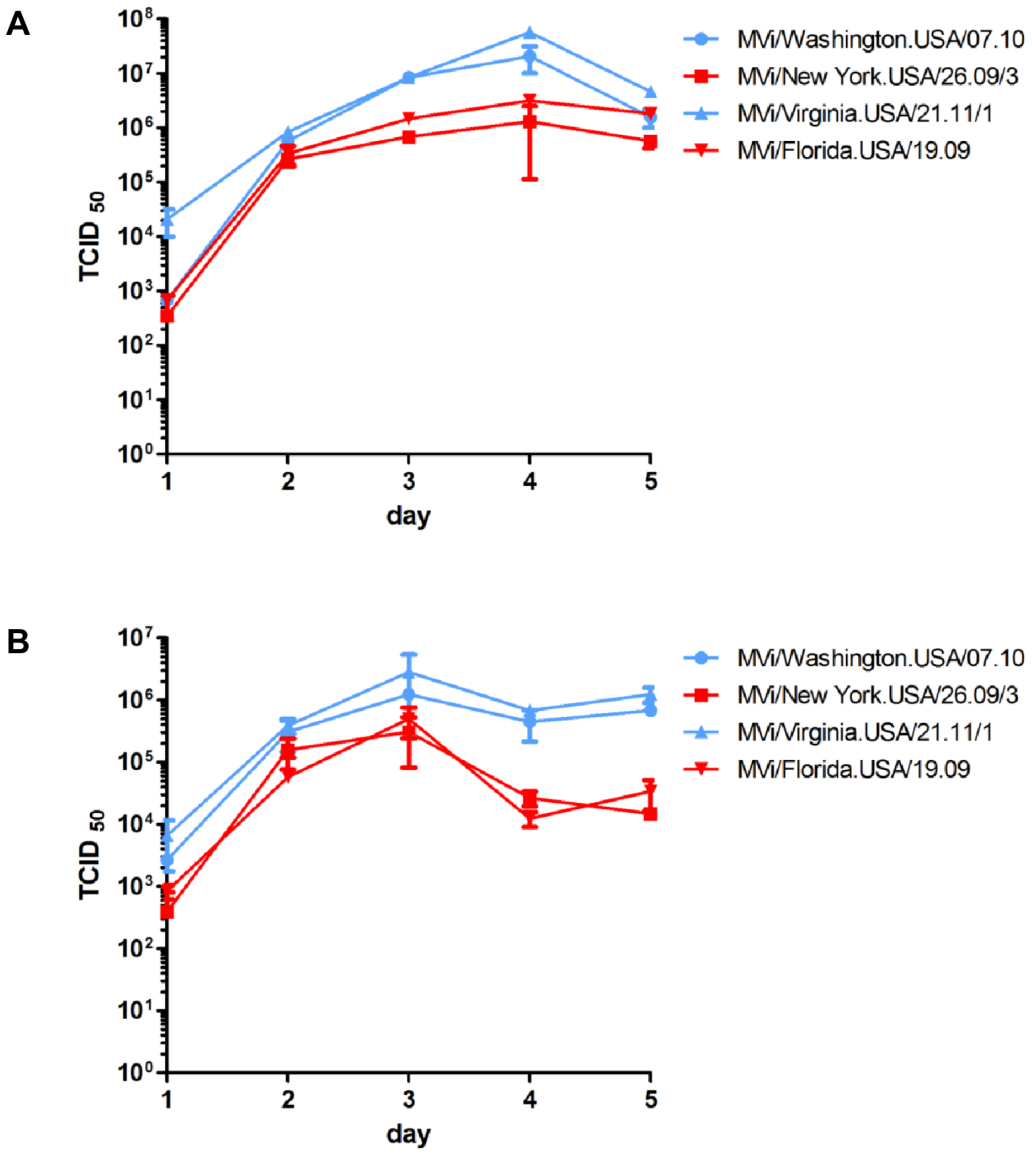
Multi-step growth curves of MeVs with and without the indels. MVi/New York.USA/26.09/3, MVi/Florida.USA/19.09, MVi/Washington.USA/07.10 and MVi/Virginia.USA/21.11/1 were used to infect Vero/hSLAM cells (A) and A549/hSLAM cells (B) at an MOI of 0.001. At the indicated time points, cells were scraped into the medium and viral titer was measured by endpoint dilution on Vero-hSLAM cells. Titers represent averages of duplicate wells. Error bars depict one standard deviation. Blue symbols and lines indicate viral isolates without indels and red symbols and lines indicate viral isolates with indels.

MeVs with indels were isolated from cases of acute measles, suggesting that the viruses are pathogenic, and these viruses were isolated in cell culture using standard methods. To compare growth characteristics in cell culture, MVi/New York.USA/26.09/3 and MVi/Florida.USA/19.09 and two viral isolates without the indel (MVi/Washington.USA/07.10 and MVi/Virginia.USA/21.11/1) were chosen for analysis. Plaque-purified viral stocks were used for multi-step growth curves in A549/hSLAM cells (human lung carcinoma cells) and Vero/hSLAM cells (African green monkey kidney cells) (Figure 3). These cell lines express the measles receptor human lymphocyte activation marker (hSLAM) required for infection by wild-type viruses [26].

**Figure 4:**
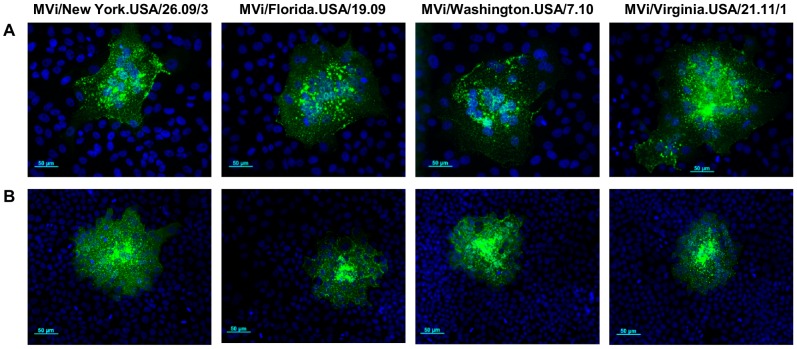
Cytopathic effect and lateral spread of genotype D4 viruses with or without indels. (A) Vero/hSLAM cells and (B) A549/hSLAM cells in chamber slides were infected with MVi/New York.USA/26.09/3, MVi/Florida.USA/19.09, MVi/Washington.USA/7.10 and MVi/Virginia.USA/21.11/1. Cells were overlaid with medium containing CMC agarose. Cells were fixed and immunostained 24 hours after infection. Representative fields are shown at 200× magnification. Green: MeV nucleoprotein, blue: nuclei.

In Vero/hSLAM cells, peak titers of the viral isolates with indels were lower than those of viral isolates without indels but final titers on day 5 were similar (Figure 3A). In A549/hSLAM cells the time course of growth was similar for days 1–3 and peak titers differed by less than one log, but final titers of the viral isolates with indels were lower than those of viral isolates without indels (Figure 3B). For both pairs of viral isolates, peak titers were higher in Vero/hSLAM cells than in A549/hSLAM cells. The ability of the four viral isolates to induce cytopathic effect in cell culture was compared in A549/hSLAM and Vero/hSLAM cells. Twenty four hours after infection, plaque sizes were approximately the same for viral isolates with and without the indels (Figure 4), demonstrating that the indels do not affect the ability to induce fusion in infected cells.

## Discussion

This is the first report demonstrating variations in genome size in wild-type MeVs. The indels were found in minimally passaged viral isolates and their presence was confirmed in the patient specimens. Sequences from 16 isolates suggested repeated, independent importation of wild type viruses with identical indels from European sources, suggesting wide-spread distribution of genotype D4 viruses with these indels in Europe.

There are 81 complete genomic sequences of MeVs available in GenBank (as of 02/18/2014), including MVi/New York.USA/26.09/3 and MVi/Florida.USA/19.09. Of these, 37 are vaccine or vaccine-related strains and 4 are from cases of subacute sclerosing panencephalitis (SSPE) (see below) [27]. The 40 remaining sequences are wild-type or derived from wild-type isolates; however, many have undergone extensive passaging in cell culture. Cell lines chosen for passaging often included non-human cell lines and/or cell lines that do not express a wild-type MeV receptor, which may lead to adaptive mutations in the genomes [28]–[30]. In contrast, MVi/New York.USA/26.09/3 and MVi/Florida.USA/19.09 are viral isolates that have undergone minimal passaging in Vero/hSLAM cells. In addition to MVi/New York.USA/26.09/3 and MVi/Florida.USA/19.09, 9 other genomic sequences deposited in GenBank showed evidence of insertions or deletions deviating from the standard genomic organization. The indels in an Edmonston-derived strain (GenBank accession number K01711, submitted in 1989), which include an insertion in the highly conserved leader sequence [31], are likely the result of sequencing errors. Three genomic sequences with indels are derived from cases of (SSPE). SSPE is a rare, fatal measles infection of the central nervous system [27]. Sequences obtained from SSPE brain tissue samples showed an accumulation of nucleotide substitutions especially in the M and F genes, often leading to reduced expression of these proteins or to expression of truncated proteins [32]–[37]. Because of these defects, most SSPE viruses remain exclusively cell-associated and do not form extra-cellular virions [27]. MVs/Zagreb.CRO/47.02 [SSPE], MVs/Zagreb.CRO/08.03 [SSPE] [38] and SSPE-Kobe-1 each have multiple indels, many of which are located in the M-F UTR. All 3 SSPE genomic sequences are 15,894 nt in length and follow the rule-of-six. Because SSPE viruses are defective, it is unclear whether the findings described above are relevant for the discussion of indels in genomes of currently circulating wild-type viruses.

The remaining 5 genomic sequences with indels are from MeVs in clade D. Four of these conserve the standard size of the measles genome. MVi/Tokyo.JPN/37.99(Y) and MVi/Tokyo.JPN/37.99(Y)C7 are genotype D3, the former is a wild-type isolate, the latter is its cell culture passaged derivative [39]. Both have identical insertions in the M 3′ UTR and identical deletions that replace 27 amino acids at the carboxyl-terminus of the F1 protein with a different sequence of 11 amino acids. MVi97-45881 and MVi/WA.USA/17.98 are wild-type isolates of genotype D6 which share identical indels in the M-F UTR [38]. Finally, MVi/Treviso.ITA/03.10/1 is a genotype D4 viral isolate that shares the same indel as MVi/New York.USA/26.09/3 and MVi/Florida.USA/19.09. A search for partial genomic sequences covering the M-F UTR found MVi/Zagreb.CRO/19.08, a genotype D4 isolate which also shows the same indels. These two sequences confirm that genotype D4 viruses with indels were circulating in Europe.

The majority of complete MeV genomes sequenced to date conform to the canonical structure. Of the few exceptions discussed here, most genomes have indels in the M-F UTR. This region is characterized by long homopolymeric sequences (Fig. 1). In DNA genomes, long homopolymeric sequences are especially prone to slippage errors during replication and transcription [40], [41]. MeV uses slippage during transcription to add poly-A tails to mRNAs and to edit the P gene transcript [42], [43]. It is possible that slippage during replication is responsible for the indels reported in the M-F UTR. A second reason for the high proportion of indels in this region may be that substitutions in coding regions are selected against if they change the amino acid sequence of a viral protein, while indels in UTRs are more likely to be maintained. MeVs utilize their genomes effectively, by expressing three different proteins from the P gene and keeping all other UTRs except the M-F UTR to 107–160 nt in length [1], [42]. Taking into account the relatively high mutation rate of RNA viruses [24], [44], it appears likely that the M-F intergenic region is conserved because it serves an important function for the virus. The M-F intergenic region is highly variable, suggesting that the function may be sequence-independent. Despite several studies with recombinant viruses, this function remains unclear. Remarkably, recombinant measles viruses with deletions of the M-F UTR or parts thereof are viable in cell culture [8], [45]. Compared to transcripts or recombinant viruses with deletions of the UTRs, presence of the F 5′ UTR modulates F protein translation initiation [7] and reduces F gene transcription, leading to decreased cytopathogenicity and lower viral titers in cell culture and in a SCID/hu mouse model [8], [46]. On the other hand, inclusion of the M 3′ UTR in recombinant viruses increases M protein production and promotes replication [8]. Our data show that viral isolates with and without indels produce similar plaque sizes and replicate efficiently in two cell lines. The higher titers in Vero/hSLAM cells may result from the inability of Vero cells to induce type I interferons [47], leading to reduced activity of antiviral innate immunity. It is difficult to reconcile the conservation of such a large UTR with the observation that it can be deleted without major effects on replication in cell culture. While the function of the M-F UTR remains unclear, the publications cited above demonstrate that changes in the length of the M-F UTR are easily tolerated, a finding that is supported by our data.

N-450 sequences of several of the viral isolates analyzed in this study are closely related to the Enfield lineage of genotype D4 that swept through Europe beginning in 2008 [48], [49]. The three viral isolates that do not share the indel, MVi/Washington.USA/07.10, MVi/Virginia.USA/20.11 and MVi/Virginia.USA/21.11/2, were imported from India, the two viral isolates from Virginia were in the same chain of transmission. Genotype D4 viruses are endemic in India with multiple co-circulating lineages present throughout the country [50]. It would be interesting to sequence related MeV isolates from Europe and India to determine whether the indels were present when the Enfield lineage was introduced into Europe and at which branch point in the phylogenetic tree the genotype D4 viruses with indels separated from those with standard genomes. Since 2013, genotype D4 has been replaced as the most frequently detected genotype in Europe and the US by genotype D8 [51].

Finding non-canonical genomes in patient samples demonstrates that whole-genome sequencing of MeV can identify unexpected deviations from the standard genome organization. This information will contribute to our understanding of the evolution and transmission patterns of MeV.

## Supporting Information

Table S1 **Primer sets designed for sequencing of measles virus.** a The first number corresponds to the starting MeV nucleotide of the forward primer and the second number corresponds to the end MeV nucleotide for reverse primer. b The first 18 nucleotides of the primer sequences are the M13 tags.
(DOCX)
