# Supplementary material for: Wild-Type Measles Viruses with Non-Standard Genome Lengths
Source: PLoS One. 2014 Apr 18;9(4):e95470. doi: 10.1371/journal.pone.0095470 (PMC3991672; doi:10.1371/journal.pone.0095470)
Supplement: Table S1 — Primer sets designed for sequencing of measles virus. a The first number corresponds to the starting MeV nucleotide of the forward primer and the second number corresponds to the end MeV nucleotide for reverse primer. b The first 18 nucleotides of the primer sequences are the M13 tags. (DOCX) [file pone.0095470.s001.docx]

| **Nucleotide numbers^a^** | **Forward Primer^b^** | **Reverse Primer^b^** |
| --- | --- | --- |
| 1-780 | TGTAAAACGACGGCCAGTACCAAACAAAGTTGGGTAAG | CAGGAAACAGCTATGACCCRGCAATYCTRTTCCTCACCA |
| 623-1423 | TGTAAAACGACGGCCAGTCARATYTGGGTCTTGCTYG | CAGGAAACAGCTATGACCCTYCTRTCTTCCTTGSCCCC |
| 1238-2027 | TGTAAAACGACGGCCAGTTCCACAYTGGCATCYGAACTC | CAGGAAACAGCTATGACCCCGCCTTCAGYTGATCCAAT |
| 59-859 | TGTAAAACGACGGCCAGTTTCAAGATCCTAYTATCAGGGACA | CAGGAAACAGCTATGACCGCAATCCTRGGTTTGTTYCCR |
| 705-1502 | TGTAAAACGACGGCCAGTCCCAACAAAGAAGGGTWGTYG | CAGGAAACAGCTATGACCGGCRGCTCTYGCATCACTTG |
| 1210-2006 | TGTAAAACGACGGCCAGTGGTDAGRAGRTCAGCTGGAAAGG | CAGGAAACAGCTATGACCGCTGWGARGCATGRTTTGCT |
| 86-883 | TGTAAAACGACGGCCAGTGCAGGATTAGRGWHATCCG | CAGGAAACAGCTATGACCGTRTCAATGTCACATATCATTTC |
| 663-1455 | TGTAAAACGACGGCCAGTACACKGCAGCTGAYTCGGAG | CAGGAAACAGCTATGACCTCTCCCTGGYTTCYCCYCG |
| 1282-2055 | TGTAAAACGACGGCCAGTGCTTGTYTCAGAGATTGC | CAGGAAACAGCTATGACCAGRTCCCTGRCCGCRGAT |
| 1485-2278 | TGTAAAACGACGGCCAGTGTGATGCRAGAGCYGCCCAT | CAGGAAACAGCTATGACCGTTCGCCAAKATCCACATCG |
| 2106-2906 | TGTAAAACGACGGCCAGTCAGGCATCAAGCACTGGGTT | CAGGAAACAGCTATGACCAGGTGTCCTTCCAGGGTRGA |
| 2726-3509 | TGTAAAACGACGGCCAGTTGATGATGAGCTGTTYTCYGATG | CAGGAAACAGCTATGACCGGTRGGTTGTATCGGAGCGA |
| 1261-2028 | TGTAAAACGACGGCCAGTTATCACDGCCGARGATGCAA | CAGGAAACAGCTATGACCACCGCCTTCAGYTGATCCA |
| 1861-2635 | TGTAAAACGACGGCCAGTTCAARGCCGAGCCCATCR | CAGGAAACAGCTATGACCTCACAMACTCGGGGACATTYC |
| 2469-3242 | TGTAAAACGACGGCCAGTAGCRCTTCCGRRACACCCAT | CAGGAAACAGCTATGACCCGCTTYCGATCCTCMTCTAGC |
| 3081-3852 | TGTAAAACGACGGCCAGTCGGACCAGTTCCAGAGGACA | CAGGAAACAGCTATGACCGGTTTGCRTTGAAGACACTCCC |
| 1230-1999 | TGTAAAACGACGGCCAGTAGGTCAGTTCCACAYTGGCATC | CAGGAAACAGCTATGACCRGCATGRTTTGCTGAGACCC |
| 1826-2602 | TGTAAAACGACGGCCAGTCCATGTCAAAAACGGRCTGG | CAGGAAACAGCTATGACCCTGGCCCTRATGGTTCCRAG |
| 2431-3205 | TGTAAAACGACGGCCAGTCTCTCAATGTTCCTCCGCCY | CAGGAAACAGCTATGACCTGGAGCGGATTACACTGCGT |
| 3053-3819 | TGTAAAACGACGGCCAGTCCGACAAMYCCARGGAATGA | CAGGAAACAGCTATGACCCCTTTCTCCAAGGTKTGAGGAGR |
| 3076-3861 | TGTAAAACGACGGCCAGTATGGACGGACCAGTTCCAGA | CAGGAAACAGCTATGACCTGCAYACTTGGTTTGCRTTGA |
| 3681-4460 | TGTAAAACGACGGCCAGTGATCYACAGCAAARCCCGAR | CAGGAAACAGCTATGACCGCKGRGCACTRCGGTCTACA |
| 4235-4952 | TGTAAAACGACGGCCAGTAGCAAGACYCTCCAYGCACA | CAGGAAACAGCTATGACCCTKGTTKTCGGGGGKTGGG |
| 3104-3904 | TGTAAAACGACGGCCAGTGCTGAAGGAATTYCARCTAAARCC | CAGGAAACAGCTATGACCCGRAACCTCTGCGGGKTRTC |
| 3741-4505 | TGTAAAACGACGGCCAGTGACGYACAGCAGGGCTCAAT | CAGGAAACAGCTATGACCCGGGCYTTCTGGYTRTCATT |
| 4163-4895 | TGTAAAACGACGGCCAGTAAGATGGGCCTGGTTTTTGC | CAGGAAACAGCTATGACCTGTGTRTGTTCCTTGGCCCT |
| 3033-3833 | TGTAAAACGACGGCCAGTCTCAAGAARCCCGTTGCCAG | CAGGAAACAGCTATGACCCCCTGTTGTTAGGACCTTTCTCC |
| 3656-4437 | TGTAAAACGACGGCCAGTGGGTYCYTGCCYTTAGGTGTT | CAGGAAACAGCTATGACCCYTTGAAYAGTCCTTGGTCATCA |
| 4066-4858 | TGTAAAACGACGGCCAGTGCARCTYCCTGAGGCAACAT | CAGGAAACAGCTATGACCCCGRGGRGRGAGGATCTGTC |
| 4235-4952 | TGTAAAACGACGGCCAGTAGCAAGACYCTCCAYGCACA | CAGGAAACAGCTATGACCCTKGTTKTCGGGGGKTGGG |
| 5003-5803 | TGTAAAACGACGGCCAGTYACCRACCCCCGAMCAGAC | CAGGAAACAGCTATGACCCCYGCAAATCTCTTGTGTCTCC |
| 5650-6432 | TGTAAAACGACGGCCAGTCCTCAAYAACTGCACRAGGGY | CAGGAAACAGCTATGACCGGTACCCTTGGGTTGCWACAT |
| 5034-5760 | TGTAAAACGACGGCCAGTCAYCGRYAATCCAAGACG | CAGGAAACAGCTATGACCCYGGTCTTATRTTYTGGGTCA |
| 5599-6371 | TGTAAAACGACGGCCAGTTCGYTCCAGCCAYCAMTCAT | CAGGAAACAGCTATGACCGTACGAGACCCCCTCWAGCC |
| 6214-6973 | TGTAAAACGACGGCCAGTTGGAGGTGATTTACTGGGCA | CAGGAAACAGCTATGACCCCAAGACACACTGCAATCAGGA |
| 5074-5870 | TGTAAAACGACGGCCAGTSCCCCMAGGGGCYGACAG | CAGGAAACAGCTATGACCAAGYGCAATGCCRGCTGTTA |
| 5707-6507 | TGTAAAACGACGGCCAGTYYTGGAACCAATTAGAGATGCAC | CAGGAAACAGCTATGACCGGTACARGGCATTTTGGCYG |
| 6253-7045 | TGTAAAACGACGGCCAGTAARGGCYCGGATAACTCACG | CAGGAAACAGCTATGACCCCAACYTGTYCTCCCTTYTTG |
| 6867-7661 | TGTAAAACGACGGCCAGTTGGAGGATGCCAAGGAAYTG | CAGGAAACAGCTATGACCTCCCTATCCGGATTRAGGAATTTR |
| 7480-8268 | TGTAAAACGACGGCCAGTTCCATAARAGCCTCAGCACCA | CAGGAAACAGCTATGACCGTCGGTTGGGGATTTCCAGA |
| 6274-7069 | TGTAAAACGACGGCCAGTCGACACAGAGTCCTACTTCATTGTM | CAGGAAACAGCTATGACCGGCTTTAGGCCTGGTCTTGA |
| 6911-7710 | TGTAAAACGACGGCCAGTAGGRGTATGAAAGGTTTRTCGAGC | CAGGAAACAGCTATGACCGATTCTCTCTGGCGGGTTGA |
| 7526-8325 | TGTAAAACGACGGCCAGTGCATCAGGTCAAGGAYGTGY | CAGGAAACAGCTATGACCGAGGTARAGCCYRTCTATCACTGG |
| 6229-7017 | TGTAAAACGACGGCCAGTGGGCATCTTAGAGAGCRGAGG | CAGGAAACAGCTATGACCGCCCCCTGCAGCAACATA |
| 6818-7600 | TGTAAAACGACGGCCAGTTTGGAGARGTTRGACGTAGGGA | CAGGAAACAGCTATGACCTCTGAGGTGTYCTCAGGCCC |
| 7428-8211 | TGTAAAACGACGGCCAGTCTRGCCATYGCAGGCATYAG | CAGGAAACAGCTATGACCTGAYCCCTGATAGGGAAYYGTG |
| 7864-8645 | TGTAAAACGACGGCCAGTTCTCAAACATGTCGYTGTCYCTG | CAGGAAACAGCTATGACCGGCGGGATAKTCAGCCAATA |
| 8485-9257 | TGTAAAACGACGGCCAGTRCAGGATTCCTTCATACGGGG | CAGGAAACAGCTATGACCGGTTGACAGAYAGCGAGTCCA |
| 9087-9869 | TGTAAAACGACGGCCAGTTGCACAGTCRMYCGGGAAG | CAGGAAACAGCTATGACCTRGCAACAAGRTCACGAGARA |
| 7823-8596 | TGTAAAACGACGGCCAGTWAAGGGRAACTGCTCAGGGC | CAGGAAACAGCTATGACCCCATCCCTGAACCGTGWGT |
| 8443-9230 | TGTAAAACGACGGCCAGTAAGCACTCTGYGARAATCCCG | CAGGAAACAGCTATGACCGRAACCRCTTGGACCCTACG |
| 9065-9845 | TGTAAAACGACGGCCAGTGATGGTGGGCATGGRAGTYA | CAGGAAACAGCTATGACCRCARCTCAACTGAACTACCAGTGAA |
| 7795-8574 | TGTAAAACGACGGCCAGTRRRCAACCAATCAGTTCCTAGCTG | CAGGAAACAGCTATGACCCAATGGMCCGAATCCYGAAG |
| 8367-9139 | TGTAAAACGACGGCCAGTGCTGTYCCGACRACACGRAC | CAGGAAACAGCTATGACCTTCACTRGCWGCCCTATCYG |
| 8975-9742 | TGTAAAACGACGGCCAGTGGAATGCTTCACATGGGAYMA | CAGGAAACAGCTATGACCAAACCAAAACAGAAAGGGYTCAA |
| 9460-10251 | TGTAAAACGACGGCCAGTCTTAGRAGTTAYCCGGCCCA | CAGGAAACAGCTATGACCATGAAAATGTAATCTARRGCWTCA |
| 10079-10856 | TGTAAAACGACGGCCAGTTGCTGGAGCCWCTTTCACTT | CAGGAAACAGCTATGACCCCTTGATCTCCTTTTCTTTCAGGC |
| 10684-11483 | TGTAAAACGACGGCCAGTTTCCTGCGTTACGAYCCTCC | CAGGAAACAGCTATGACCTGATGGTCCACAGCTTCTGACA |
| 9439-10217 | TGTAAAACGACGGCCAGTGGGAATGTCATCAAGTCYAA | CAGGAAACAGCTATGACCGATAAGTAYCTTCATCAGAAAAY |
| 10032-10821 | TGTAAAACGACGGCCAGTTGGTTTCTTCCCTGCACTCG | CAGGAAACAGCTATGACCTTGAACTCAGGGTCATGGAGG |
| 10629-11429 | TGTAAAACGACGGCCAGTGGACAAGGCACTTGCTGCTC | CAGGAAACAGCTATGACCTGAAGATTTGRTCATTGGGGA |
| 9373-10159 | TGTAAAACGACGGCCAGTCAGAACATCAAGCACCGCCT | CAGGAAACAGCTATGACCGCAGTGGTTAAGGAAAGCACCTC |
| 9967-10765 | TGTAAAACGACGGCCAGTATGACYATTGATGCTAGGTATRCWG | CAGGAAACAGCTATGACCTGGGTCAAAGCTMGAATCAY |
| 10589-11374 | TGTAAAACGACGGCCAGTTGCCTCTTAGCCTGGAYAGTG | CAGGAAACAGCTATGACCGGGGCAATGAGGRTCACTYA |
| 11053-11849 | TGTAAAACGACGGCCAGTGGGCCAGTCYYAAAAACCYA | CAGGAAACAGCTATGACCCRATATTRCTGCATGCTGCCC |
| 11670-12449 | TGTAAAACGACGGCCAGTTGAYATTGGCCATCACCTCAA | CAGGAAACAGCTATGACCTTGCCCCTGTGACACTRTGR |
| 12284-13064 | TGTAAAACGACGGCCAGTCTGCAAGGTTTGTCYTRRTCCA | CAGGAAACAGCTATGACCTYACCCTTAGCTCCTCCAGGC |
| 11014-11810 | TGTAAAACGACGGCCAGTTCAGGAGTCCCCAAAGATCTCA | CAGGAAACAGCTATGACCTCTCTGACCARAATACACAYCTTGC |
| 11644-12427 | TGTAAAACGACGGCCAGTTTYGTAATTCTTAGGCAAAGGC | CAGGAAACAGCTATGACCYAGGATYTCATGAGCTGCCCTA |
| 12240-13040 | TGTAAAACGACGGCCAGTTCTYGTATGYGTCCAGAGCATCA | CAGGAAACAGCTATGACCCATTGGCYCTTTGCCTDGC |
| 10992-11783 | TGTAAAACGACGGCCAGTGGCACTCCACACTCTRGCTG | CAGGAAACAGCTATGACCTGCTCTTGAGTGATTGGGACA |
| 11608-12391 | TGTAAAACGACGGCCAGTAAGAAAYGGGAAGCTGCYAGA | CAGGAAACAGCTATGACCCCTGTCCATGAGGAATGCMG |
| 12206-12993 | TGTAAAACGACGGCCAGTTCCTRGACTGGGCTAGYGACC | CAGGAAACAGCTATGACCTCATCATCACCSKAAGCCCA |
| 12618-13393 | TGTAAAACGACGGCCAGTYAARGAGTCATGYTCAGTGCAGC | CAGGAAACAGCTATGACCTAGCTTGCGRGAGCTGGGTA |
| 13233-14028 | TGTAAAACGACGGCCAGTCCARCAARGAATGCTYCTAGGG | CAGGAAACAGCTATGACCTCCTCRTCGCTTTCRCACAR |
| 13877-14677 | TGTAAAACGACGGCCAGTTAGAGCCTATCCAYGGTCCT | CAGGAAACAGCTATGACCGGAAACCCCACTATTRTAGAAGCA |
| 12571-13355 | TGTAAAACGACGGCCAGTGCAGGGATGGTGCTATTGACA | CAGGAAACAGCTATGACCTCGGGATCACGCAACAATCT |
| 13167-13967 | TGTAAAACGACGGCCAGTCACAATCTCCAAYGACAATCTCTCA | CAGGAAACAGCTATGACCCRAGGTAGGTCATATAGCATGTGT |
| 13799-14598 | TGTAAAACGACGGCCAGTGCAAAGGRGTGTTTAARGTGCTT | CAGGAAACAGCTATGACCCCCAAGAACAAGCCGTCYTC |
| 12494-13160 | TGTAAAACGACGGCCAGTGAGCCAGCATGARRAARGGG | CAGGAAACAGCTATGACCTYGCCACTCKGACAAGGGAK |
| 13000-13783 | TGTAAAACGACGGCCAGTTGGAACGAAGCCTGGTTGYT | CAGGAAACAGCTATGACCYGADGACAACAGCTCACCCA |
| 13612-14406 | TGTAAAACGACGGCCAGTTCAGCTCTMATAGGRGATGACGA | CAGGAAACAGCTATGACCTCATCGTGTGGRGGTCTGAA |
| 14226-15016 | TGTAAAACGACGGCCAGTAGACCATTACTCATGCTCYCTGACY | CAGGAAACAGCTATGACCCTGAACAAARTCCCCGCTGA |
| 14856-15622 | TGTAAAACGACGGCCAGTTGTGGGRTTTATCCAYTCHG | CAGGAAACAGCTATGACCYAGATARCCGGACTTRAGATTC |
| 15324-15895 | TGTAAAACGACGGCCAGTYTGYGGGYTGGCAATTAACG | CAGGAAACAGCTATGACCCCAGACAAAGCTGGGAATAGAA |
| 14197-14994 | TGTAAAACGACGGCCAGTTCTTCGTGGAACATAAATCCAA | CAGGAAACAGCTATGACCGGCATAAGCTTRATCACYAGTATTG |
| 14789-15589 | TGTAAAACGACGGCCAGTCYGAAGTYACRTGGGTAGGCA | CAGGAAACAGCTATGACCATTWATCAACTTTYTGTTCC |
| 15176-15819 | TGTAAAACGACGGCCAGTCTGTGMGGACYTCACCTGGA | CAGGAAACAGCTATGACCRGGGYAGGATTAGGGTTCCG |
| 14169-14962 | TGTAAAACGACGGCCAGTGGMAGAGGCTAKGYTATCTCCAGC | CAGGAAACAGCTATGACCTTTGCCMARGAGYAGAGCCA |
| 14766-15563 | TGTAAAACGACGGCCAGTCAARGTGCTCTTTAACGGGAGG | CAGGAAACAGCTATGACCAAAGAAGAATRTGCCCCCARA |
| 15114-15776 | TGTAAAACGACGGCCAGTWGAYCTCAARGCTAACCGGC | CAGGAAACAGCTATGACCTCAGRGCRCTGTATCCGACT |
